# Protocol to Establish Estrogen Receptor-Negative Heterozygous BRCA1 Organoids

**DOI:** 10.3390/mps8060127

**Published:** 2025-11-01

**Authors:** Madhura Deshpande, Jeannine Gerhardt

**Affiliations:** 1 The Ronald O. Perelman and Claudia Cohen Center for Reproductive Medicine, Weill Cornell Medicine, New York, NY 10021, USA; 2 Department of Obstetrics and Gynecology, Weill Cornell Medicine, New York, NY 10021, USA

**Keywords:** β-Estradiol-receptor-negative cells, human organoids, BRCA germline mutation

## Abstract

Cancer development in BRCA1 carriers is a multi-step process, which is triggered by several factors and mechanisms that are not clearly understood. Most BRCA1 carriers develop triple-negative breast cancer (TNBC)—estrogen receptor (ER)-, progesterone receptor (PR)-, and HER2 -negative cancers—which originates from ER/PR/HER2-negative breast progenitor cells. Due to a lack of ER/PR/HER2-negative cell models with BRCA mutations, the processes inducing cancer development in BRCA carriers have not been comprehensively studied. Thus, studies characterizing ER/PR/HER2-negative cells carrying a BRCA1 germline mutation are needed to gain more in-depth knowledge about the steps leading to cancer initiation in BRCA1 carriers. To study the cancer development in these patients, we established a protocol for the generation of human ER/PR/HER2-negative breast organoids carrying a BRCA1 germline mutation. We confirmed that these organoids are unresponsive to estrogen, can self-renew, and express the stem/progenitor marker CD44. In addition, we observed that these organoids contain outgrowths that resemble the mature ductal and lobular units of the mammary gland, thus making it a suitable model system to study how cancer develops in ER/PR/HER2-negative mammary cells that carry a BRCA1 germline mutation.

## 1. Introduction

Germline mutations at the BRCA1 gene are associated with an increased risk of breast, ovarian, and prostate cancers, as well as other types of malignancies [1]. Cancer development in carriers with a BRCA1 variant is a multi-step process that is triggered by several factors and defects, which are not clearly understood. To prevent cancer development and identify novel preventive strategies for BRCA1 variant carriers, it is essential to understand the precise mechanisms and factors that cause carcinogenesis in these patients. BRCA1 patients carry a germline mutation at one of the BRCA1 alleles. During cancer development, mutations in the other BRCA allele are induced in 90% of BRCA cancer cells, leading to locus-specific loss of heterozygosity (LOH) [1,2,3,4]. BRCA1 is a tumor-suppressor gene and a key player in several cellular pathways, including DNA repair, gene transcription, cell differentiation, chromatin structures, and cell cycle regulation, which are defective if BRCA1 gene function is lost. However, it is unclear which of the multiple functions of BRCA1 are completely defective in heterozygous BRCA1 carrier cells, which have only one mutated BRCA allele. It was shown that some of the functions, such as DNA repair by HR, are not affected in heterozygous BRCA1^mut/+^ cells [5]. In contrast, the repair of stalled replication forks was found to be defective in these cells. In addition, we recently discovered an upsurge in error-prone DNA repair in BRCA1^mut/+^ cells that causes mutations and LOH [6].

To understand more clearly how genomic instability and mutations in heterozygous BRCA1 cells lead to cancer initiation and identify factors that facilitate this development, there is a need to establish better cell models that more closely resemble the cells in BRCA1 variant carriers. So far, most BRCA studies use animal models, 2D cell cultures, or human organoids generated from cancerous tissue. These cells do not exactly resemble the heterozygous cells in BRCA1 carriers, because most BRCA cancer cells show LOH and other genomic alterations. In addition, 3D human organoids resemble the cells in the human body more closely than 2D cell culture and are progressively used to study cancer development. Furthermore, 70% of BRCA1-associated breast cancers are triple-negative breast cancer (TNBC): estrogen receptor (ER)-, progesterone receptor (PR)-, and HER2-negative cancers [7]. It was recently proposed that BRCA1 triple-negative cancer cells may originate from ER/PR/HER2-negative breast stem/progenitor cells that do not express ER; however, loss of ER expression could also occur during anomalous cell development [8,9]. So far, the processes leading to cancer development in ER-positive cells have been extensively studied, but not yet in ER-negative cells. Thus, BRCA1 cell models that are ER- and PR-negative are needed to gain more in-depth knowledge about the steps leading to cancer in ER-negative cells. To study the cancer development in ER/PR/HER2-negative cells carrying a BRCA1 germline mutation, we established a protocol for the generation of human BRCA1 organoids from ER/PR/HER2-negative heterozygous BRCA1 cells.

## 2. Experimental Design

### 2.1. Cell Lines

The mammary cell line MCF10A is an ER/PR/HER2-negative cell line that expresses luminal, stem, and progenitor cell markers [10,11]. MCF10A is a suitable 2D cell line to establish ER-negative heterozygous BRCA1 organoids to analyze the mechanisms leading to TNBC initiation. We used two heterozygous BRCA1 MCF10A cell lines, which were generated, published, and gifted to us by Dr. Ben Ho Park [12]. The cell lines are also sold by Horizon Discovery.

- BRCA1.1 MCF10A—heterozygous BRCA1 mammary cell line, which has the 185delAG mutation (Founder mutation) in one allele.
- BRCA1.2 MCF10A—heterozygous BRCA1 mammary cell line, which has the R71G mutation (Arg to Gly change at codon 71) in one allele.

### 2.2. Materials

- Greiner Bio-One CELLSTAR cell culture multi-well plates for suspension cultures, 6-well (Fisher Scientific, Cat #07-000-646, Waltham, MA, USA)
- Atrazine (Sigma Aldrich, Cat #49085, St Louis, MI, USA)
- Cholera toxin (Sigma Aldrich, Cat #C-8052, St Louis, MI, USA)
- DMEM/F12 (ThermoFisher Scientific Cat #11330-032, Waltham, MA, USA)
- Epidermal growth factor (EGF, Peprotech, Cat# AF-100-15, Cranberry, NJ, USA)
- Ethylene glycol tetraacetic acid (EGTA-pH 8 Alfa Aesar, Cat #J60869, Lancashire, UK)
- Estrogen (β-estradiol, Sigma Aldrich, Cat #E2758, St Louis, MI, USA)
- Human embryonic stem cells (hESC) Matrigel (Corning, Cat #354277, Charlotte, NC, USA)
- Horse serum (ThermoFisher Scientific, Cat #16050-122, Waltham, MA, USA)
- Hydrocortisone (Sigma Aldrich, Cat #H088, St Louis, MI, USA)
- Insulin (Sigma Aldrich, Cat #I-1882, St Louis, MI, USA)
- Matrigel, growth factor reduced (Corning, Cat # 356231, Charlotte, NC, USA)
- Matrigel, organoid (Corning, Cat # 356255, Charlotte, NC, USA)
- 1 M magnesium chloride (MgCl_2_ Sigma Aldrich, Cat #M1028, St Louis, MI, USA)
- Paraformaldehyde (Merck, Cat #1004960700, Rahway, NJ, USA)
- Phosphate-buffered saline (Sigma, Cat #D8537, St Louis, MI, USA)
- Penicillin-streptomycin (ThermoFisher Scientific, Cat #15070063, Waltham, MA, USA)
- PIPES buffer (pH 6.8) (Alfa Aesar, Cat #J60300, Lancashire, UK)
- ProLong Gold antifade mountant with DAPI (ThermoFisher Scientific, Cat #P36935, Waltham, MA, USA)
- TrypLE (ThermoFisher Scientific, Cat #12604013, Waltham, MA, USA)
- Triton X-100 (Sigma Aldrich, Cat #X100, St Louis, MI, USA)

### 2.3. Equipment

- Biosafety cabinet (various manufacturers)
- 37 °C incubator with 5% CO_2_ (various manufacturers)
- Echo brightfield microscope
- Confocal microscope (various manufacturers)

## 3. Procedure

### 3.1. Cell Culture Maintenance

1. Place complete growth medium in a 37 °C water bath to warm up.
2. Add 9 mL of media (DMEM/F12 supplemented with 5% horse serum, 20 ng/mL epidermal growth factor, 10 μg/mL insulin, 0.5 μg/mL hydrocortisone, 0.1 μg/mL cholera toxin, and 1% penicillin-streptomycin) to a new 15 mL conical tube.
3. Remove the cell lines from liquid nitrogen storage.
4. Thaw the cell lines by swirling the tubes in a 37 °C water bath for 3–5 min. The tubes were kept at 37 °C until the suspension was completely thawed. Remove the tubes immediately.
5. Gently mix the cells by tapping and transfer to a 15 mL tube.
6. Gently mix by pipetting.
7. Centrifuge the suspension at 125 g for 10 min.
8. Aspirate the supernatant gently.
9. Add 10 mL fresh medium and mix gently. Collect an aliquot for cell count.
10. Plate the cells in pre-labeled T-75 flask at about 2.5 × 10^4^ cells/cm^2^.
11. Incubate at 37 °C in 5% CO_2_ incubator.
12. Observe cells daily. Change media every 2 days.
13. Confluent cells are observed after 4–5 days. Use these to generate organoids.

### 3.2. Initial Organoid Establishment (Figure 1)

1. Thaw Matrigel on ice.
2. Pre-warm low attachment 6-well plates at 37 °C.
3. Harvest confluent cells and determine cell count.
4. Adjust the cell count to 1 × 10^4^ cells/drop (50 µL), i.e., 2 × 10^5^ cells/mL. Keep the cell suspension on ice.
5. Add an equal volume of cold Matrigel to the cell suspension (Media:Matrigel ratio 1:1)
6. Mix well gently but avoid introducing any air bubbles.
7. Gently drop 50 µL of the cell suspension (containing 0.5–1 × 10^4^ cells for each well) to pre-warmed low attachment 6-well plates.
8. Incubate the plates at 37 °C for 30 min to allow the Matrigel dome to solidify.
9. Add 2 mL of pre-warmed media to the well. Ensure that the domes are solidified.
10. Additions to the media can be made to check the effect of the compounds on organoid formation. In our study, we exposed the organoids to estradiol or atrazine and replaced the media every 2–3 days.
11. Incubate the plate at 37 °C in 5% CO_2_ incubator.
12. Observe the organoids and record the morphology.
13. The images of the organoids were taken using an Echo brightfield microscope on days 0, 2, 4, 6, 8, and 10. The media was changed every 2–3 days.
14. After 10–15 days, the organoids were harvested for passaging as well as for staining.

**Figure 1:**
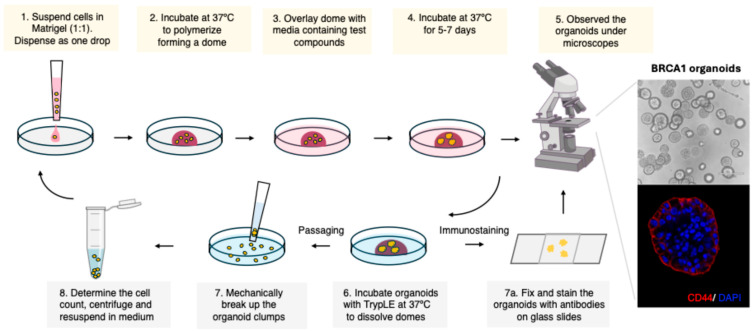
Steps for the establishment and passaging of human ER/PR/HER2-negative breast organoids carrying a BRCA1 germline mutation (BRCA1^mut/+^ breast organoids).

### 3.3. Passaging Organoids (This Step Is Performed Every 10–15 Days, Depending on the Growth Rate and Concentration of the Organoids per Well)

1. Thaw Matrigel on ice.
2. Pre-warm low attachment 6-well plates at 37 °C.
3. Remove media from the organoid plate. Wash with cold PBS.
4. Add cold TrypLE and incubate for 1-3 min at RT.
5. Add 1 mL of cold media and use large-bore tips (You can cut a 1 mL tip) to break the clumps
6. Do not over pipett. Ensure that you see cell fragments as well under the microscope.
7. Add 5 mL of cold media and centrifuge.
8. Remove the supernatant and add cold media; then keep the mixture on ice.
9. Determine live cell count.
10. Adjust the cell count to obtain 1 × 10^4^ single cells or small fragments/drop (50 µL).
11. Add the cells to cold Matrigel-Media mix (1:1 ratio).
12. Mix well but avoid introducing any air bubbles.
13. Gently drop 50 µL of the cell suspension (containing 0.5–1 × 10^4^ cells for each well) to pre-warmed low attachment 6-well plates.
14. Incubate the plates at 37 °C for 30 min to allow the Matrigel dome to solidify.
15. Add 2 mL of pre-warmed media to the well. Ensure that the domes are solidified.
16. Incubate the plate at 37 °C in 5% CO_2_ incubator.
17. Observe the organoids and record the morphology.
18. The images of the organoids were taken using an Echo brightfield microscope on days 0, 2, 4, 6, 8, and 10. The media was changed every 4 days.
19. After 10–15 days, the organoids were harvested for passaging as well as for staining.

### 3.4. Immunostaining of Organoids

1. Thaw Matrigel on ice.
2. Pre-warm low attachment 6-well plates at 37 °C.
3. Remove media from the organoid plate. Wash with cold PBS.
4. With a large tip, pipette gently to dislodge the dome. Try to extract the organoids detached from Matrigel.
5. Add 5 mL cold PBS and transfer to a 50 mL tube. Centrifuge.
6. Remove supernatant, add cold PBS, and repeat the wash.
7. Depending upon the experimental set-up, you can aliquot the organoids into individual tubes and proceed.
8. Transfer the organoids to a microfuge tube (for ease of handling) and centrifuge to remove PBS.
9. Fix the organoids with the PMTEF buffer for 30–40 min at RT (4% paraformaldehyde, 200 mM PIPES-pH 6.8, 200 mM MgCl_2_, 10 mM EGTA, 0.2% Triton X).
10. Wash gently with PBS and block with 1% BSA and 0.3% Triton X in PBS (PBST) for 1 hr.
11. Remove the blocking buffer by centrifugation and incubate overnight with primary antibody at 4 °C.
12. The next day, remove the primary antibody solution and wash thrice with PBS.
13. Following PBS wash, incubate the organoids with secondary antibody (Alexa 594; 1:1000 in PBST) at room temperature for 1 hr.
14. Wash thrice with PBS and gently drop the organoids on charged slides.
15. Mount with ProLong Gold antifade mountant with DAPI.
16. The images can be taken using an immunofluorescence or confocal microscope. In the present study, an NL5 confocal microscope was used for imaging.

**Remarks** **1.** *Primary antibodies used in immunofluorescence studies were CD44 (CD44 antibody [Hermes-3], Mouse mAB #ab254530 Abcam, 1:500, Cambridge, UK) and estrogen receptor alpha F-10 antibody (Mouse mAB, Santa Cruz Biotechnology, Dallas, TX, USA, sc-8002, 1:100).*

## 4. Expected Results and Discussion

**Figure 2:**
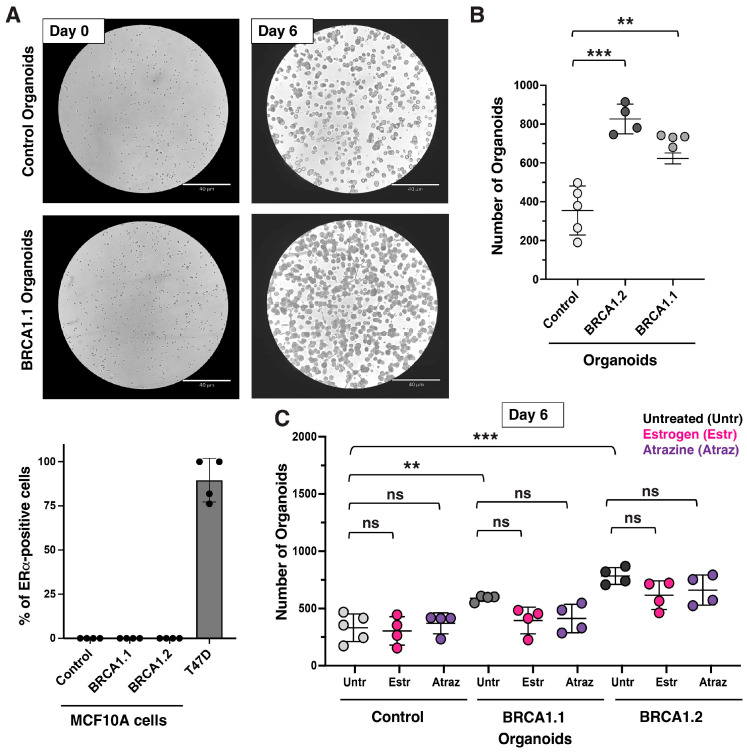
BRCA1^mut/+^ breast organoids with two different germline mutations were established (BRCA1.1 has a 185delAG and BRCA1.2 has a R71G mutation). (**A**) **Top**; 10^4^ cells/well were seeded for the generation of the breast organoids. **Bottom**; Analysis of ER alpha (ERa) expression in control and BRCA1 MCF10A cells, and T47D cancer cells (n>100). (**B**) After 6 days, the organoids were counted (two independent experiments). Statistical analysis was conducted using Student’s *t*-test with two-tailed distribution for *p*-value calculation. BRCA1^mut/+^ breast organoids had a higher proliferation rate, evident by an increased number of organoids after an equal number of cells were seeded at day 0. (**C**) Organoids were incubated with β-estradiol (Estr) and atrazine (Atraz), an endocrine-disrupting pollutant. The organoid formation on day 6 was determined by counting the organoids from images from two independent experiments. Statistical analysis was conducted using Student’s *t*-test with two-tailed distribution for *p*-value calculation. Control and BRCA1^mut/+^ breast organoids are unresponsive to both.

**Figure 3:**
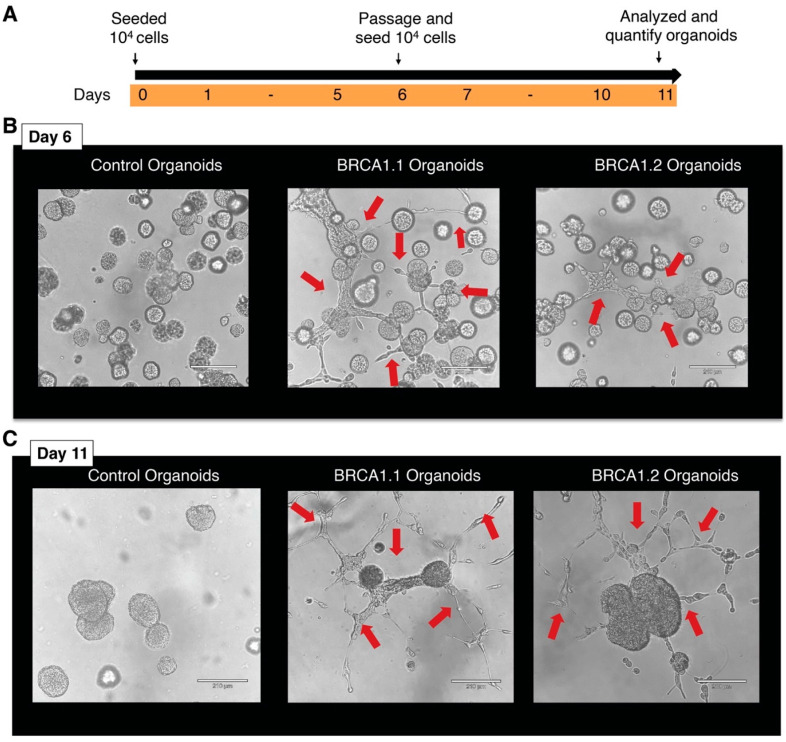
BRCA1^mut/+^ breast organoids exhibit ductal outgrowth and branches. (**A**) Schematic representation of the timeline used for the establishment of organoids. (**B**) Ductal outgrowth (red arrows) was detected in BRCA1^mut/+^ breast organoids on day 6. (**C**) After passaging, ductal outgrowth was detected in BRCA1^mut/+^ breast organoids post-day 11.

Using the described protocol (Figure 1), we successfully generated organoids from isogenic ER-negative control (wildtype, BRCA1^+/+^) and two ER-negative BRCA1^mut/+^ MCF10A cell lines that carry a BRCA1-founder mutation (BRCA1.1 and BRCA1.2, Figure 2A). An equal number of cells were seeded on day 0. After 6 days, organoids were examined, and the number of cells was analyzed. Organoids can self-assemble from single cells in a scaffolding extracellular environment (such as Matrigel) and can regenerate, as we have shown for the BRCA1 organoids (Figure 2A,B and Figure 3C). Thus, organoids slightly differ from spheroids, but the names are sometimes used interchangeably. All organoids had similar sizes (diameter) and shapes. However, the number of BRCA1^mut/+^ organoids was greater than that of control organoids (Figure 2A,B), suggesting that BRCA1^mut/+^ organoids have a higher clonogenicity than control organoids. It was previously reported that BRCA1 organoids derived from patient samples had an increase in the percentage of luminal progenitor cells (~15–20%) [13]. A higher number of breast stem/progenitor cells in the cell population could explain the generation of a higher number of organoids. MCF10A is an ER/PR/HER2-negative cell line that expresses luminal, stem, and progenitor cell markers [10,11]. β-estradiol is known to increase the cell proliferation rate. To confirm that organoids are ER/PR/HER2-negative, we treated organoids with β-estradiol and analyzed the proliferation rate. As expected, after exposure to 1 μM β-estradiol (for 6 days), the proliferation rate in these ER-negative control and BRCA1^mut/+^ organoids did not change (Figure 2C). In addition, we incubated organoids with atrazine (concentration: 1 μg/mL) for 6 days. Atrazine is known for activating aromatase activity and increasing the β-estradiol level in human cells. Atrazine is an herbicide used for weed control in crops and found in tap water. Similarly, after exposure to atrazine, we detected no alteration in the proliferation rate of control and BRCA1^mut/+^ organoids (Figure 2C).

Next, we examined the organoids more closely and observed that the organoids had outgrowths that resembled the mature ductal and lobular units of the breast (Figure 3A,B). We detected the outgrowths in untreated BRCA1^mut/+^ breast organoids at day 6–7. Human mammary luminal cells were reported to give rise to complex branched ductal structures in collagen type I gels with the addition of collagen IV [14]. Surprisingly, we observed outgrowths without supplementing additional collagen IV to the Matrigel (Figure 3B). We tested several Matrigel and found that outgrowths were seen in BRCA1^mut/+^ breast organoids incubated in different Matrigel, such as growth factor-reduced Matrigel and hESC Matrigel. Both types of Matrigel have similar base composition. The reconstituted basement membrane in the Matrigel was extracted from the Engelbreth-Holm-Swarm (EHS) mouse sarcoma that was enriched with extracellular matrix proteins, and contained 60% laminin, 30% collagen IV, and 8% entactin. Although the Matrigel also contained heparan sulfate proteoglycan (perlecan), transforming growth factor (TGF-beta), epidermal growth factor (EGF), insulin-like growth factor (IGF-1), fibroblast growth factor (bFGF), tissue plasminogen activator, and other growth factors that occur naturally in the EHS tumor, the concentration of these factors varied in different types of Matrigel. The growth factor-reduced Matrigel had lower concentrations of growth factors. It is also important to note that the standard formulation of Matrigel can have batch-to-batch variation in the concentrations of growth factors. We also found the same phenotype after passaging, where the organoids are re-established from single cells (Figure 3C). We observed that BRCA1^mut/+^ organoids had more ductal outgrowths than control organoids.

**Figure 4:**
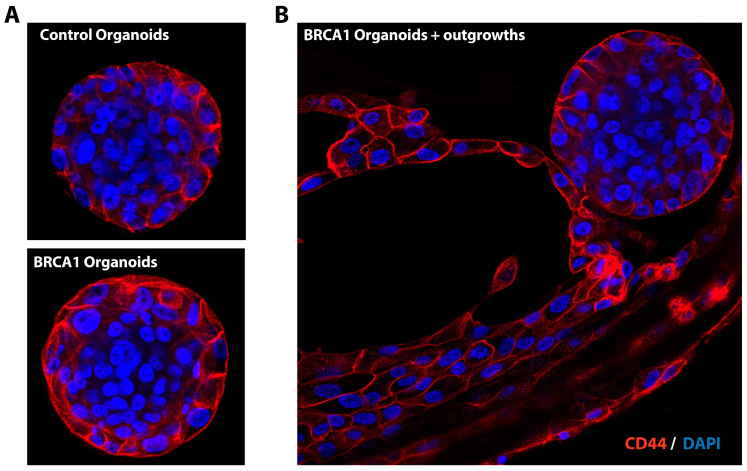
Immunostaining of breast organoids with stem cell marker CD44. (**A**) Isogenic control and BRCA1^mut/+^ breast organoids were stained with CD44 antibody (red). Nuclei were stained with DAPI (blue). CD44 was found in control and BRCA1^mut/+^ breast organoids at the cell periphery. (**B**) CD44 staining of organoids with outgrowths.

It was reported that MCF10A cell lines contained a population of mammary stem and progenitor cells [10,11], explaining the self-renewal capacity of the organoids after passaging (Figure 3C) and differentiation potential into the mammary branches and outgrowths (Figure 3B). To confirm the existence of mammary stem and progenitor cells, we stained the mammary organoids with the progenitor/stem cell marker CD44 [15,16]. CD44 is a glycoprotein engaged in cell–cell interactions and needed for various cellular functions like adhesion, migration, and signaling. As expected, we detected CD44 at the cell periphery in control and BRCA1^mut/+^ organoids (Figure 4A,B). Thus, enrichment of the progenitor/stem cell subpopulation in the MCF10A cell lines using FACS and stem cell markers could be used to increase the number/yield of BRCA1 organoids. In summary, we generated organoids from heterozygous BRCA1 cell lines, which could be used for future studies into the mechanisms and factors triggering cancer initiation and progression. Studying ER/PR/HER2-negative heterozygous BRCA1^mut/+^ organoids will give us a better understanding of the steps leading to TNBC development and neoplastic growth in the breast tissue of BRCA1 carriers. This knowledge will help us to develop novel strategies to prevent cancer development in BRCA1 carriers.

## Data Availability

The original contributions presented in this study are included in the article material. Further inquiries can be directed to the corresponding authors.
